# Prostatic Alpha-Linolenic Acid (ALA) Is Positively Associated with Aggressive Prostate Cancer: A Relationship Which May Depend on Genetic Variation in ALA Metabolism

**DOI:** 10.1371/journal.pone.0053104

**Published:** 2012-12-28

**Authors:** Maria Azrad, Kui Zhang, Robin T. Vollmer, John Madden, Thomas J. Polascik, Denise C. Snyder, Mack T. Ruffin, Judd W. Moul, Dean Brenner, Robert W. Hardy, Wendy Demark-Wahnefried

**Affiliations:** 1 Department of Nutrition Sciences, University of Alabama at Birmingham, Birmingham, Alabama, United States of America; 2 Department of Pathology, Duke University Medical Center, Durham, North Carolina, United States of America; 3 Department of Surgical Pathology & Cytopathology, Durham Veterans Affair Medical Center, Durham, North Carolina, United States of America; 4 Department of Surgery and Duke Prostate Center, Duke Cancer Institute, Duke University Medical Center, Durham, North Carolina, United States of America; 5 Department of Clinical Research, Duke University Medical Center, Durham, North Carolina, United States of America; 6 Department of Family Medicine, University of Michigan, Ann Arbor, Michigan, United States of America; 7 Department of Internal Medicine, University of Michigan, Ann Arbor, Michigan, United States of America; 8 Department of Pathology, University of Alabama at Birmingham, Birmingham, Alabama, United States of America; IPO, Inst Port Oncology, Portugal

## Abstract

Previous observational studies have reported associations between prostate cancer and alpha-linolenic acid (ALA). However, few investigations have been able to study this relationship prospectively and in well-controlled settings. Moreover, no studies have determined whether single nucleotide polymorphisms (SNPs) that influence ALA metabolism are associated with this common cancer. The purpose of this study was to explore associations between prostatic levels of ALA, SNPs and prostate cancer-specific biomarkers in samples collected from a previous randomized clinical trial conducted using a presurgical model and which tested the effects of flaxseed supplementation, a rich source of ALA, prior to prostatectomy (n = 134). Serum prostate-specific antigen (PSA) was determined and immunohistochemistry was used to assess tumor proliferation rate (Ki67). Prostatic ALA was determined with gas chromatography. Seven previously identified SNPs associated with delta-6 desaturase activity (rs99780, rs174537, rs174545, rs174572, rs498793, rs3834458 and rs968567) were tested for associations with prostatic ALA, PSA and Ki67. Despite consuming seven times more ALA per day, men in the flaxseed arm had similar amounts of prostatic ALA relative to men not consuming flaxseed. In unadjusted analysis, there were significant positive associations between prostatic ALA and PSA (ρ = 0.191, p = 0.028) and Ki67 (ρ = 0.186, p = 0.037). After adjusting for covariates (flaxseed, age, race, BMI and statin-use) the association between ALA and PSA remained (p = 0.004) but was slightly attenuated for Ki67 (p = 0.051). We did not observe associations between any of the SNPs studied and prostatic ALA; however, in models for PSA there was a significant interaction between rs498793 and ALA and for Ki67 there were significant interactions with ALA and rs99780 and rs174545. Independent and inverse associations were observed between rs174572 and Ki67. This study provides evidence that prostatic ALA, independent of the amount of ALA consumed, is positively associated with biomarkers of aggressive prostate cancer and that genetic variation may modify this relationship.

## Introduction

One out of six American men will be diagnosed with prostate cancer during their lifetime, and each year over 33,000 men die of this disease [1]. The factors which separate indolent from aggressive disease remain unknown. Because prostate cancer is more prevalent in Western societies, it is hypothesized that both genetic and environmental factors play a prominent role in its etiology. Diet is considered one of the major modifiable environmental factors influencing disease course [2].

Dietary intake of omega-3 polyunsaturated fatty acids (PUFAs) is proposed to be associated with the pathogenesis and progression of prostate cancer [3]. While the 20 carbon eicosapentaenoic acid (EPA) is considered to be protective [4], its 18 carbon precursor, alpha-linolenic acid (ALA), has been linked with increased risk for prostate cancer in some (but not all) studies [5], [6]. Given the inconsistent results from epidemiological studies, a meta-analysis of 16 studies concluded that there is a lack of a significant association between dietary intake of ALA and risk for prostate cancer [7]. Interestingly, the meta-analysis found that higher physiological levels of ALA in sera, erythrocytes or adipose tissue, were associated with 54% increased risk for prostate cancer [7]. The discordance between dietary ALA and prostate cancer risk and physiological levels of ALA and prostate cancer may be a function of the difficulties in collecting accurate dietary data. However, the discordance may be related to variation in the metabolism of ALA.

Tissue levels of ALA are in part dependent on dietary intake. Also, delta-6 desaturase, the desaturase enzyme that catalyses the rate-limiting step in ALA metabolism determines tissue levels of ALA. This enzyme is expressed mainly in the liver but in other organs, including the prostate, and dietary intake of PUFAs has been shown to regulate its expression in tissue [8]. Furthermore, dietary linoleic acid (LA) requires delta-6 desaturase for biosynthesis of arachidonic acid and thus competes with ALA for desaturase [8]. Thus a higher LA to ALA ratio, such as that seen in a Western diet, results in a shift that favors LA and hinders ALA metabolism [9]. In addition, genetic variation plays a major role in ALA metabolism. Single nucleotide polymorphisms (SNPs) in and near *FADS2*, the gene which encodes delta-6 desaturase, have been highly associated with levels of ALA in erythrocytes, plasma and serum in previous population genetic studies [10], [11] and comprehensive genome-wide association studies [12], [13]. Studies have shown that the presence of the minor allele in several SNPs is significantly associated with higher blood levels of ALA [10]–[13]. The strong and consistent associations reported for SNPs in this genetic region indicate that genetic variation alters delta-6 desaturase activity, functionality, or expression, resulting in modified ALA metabolism [14]. The role that genetic variation plays in prostatic tissue levels of ALA and prostate cancer is currently unknown but warrants investigation [15].

To date, there have been no clinical trials that have investigated the effects of dietary ALA supplementation in men with prostate cancer [7]. However, in our previous phase II randomized clinical trial (RCT), diets that were supplemented with 30 g/d of flaxseed and provided 6.51 g/d of ALA, were tested against diets with normal ALA intake in men diagnosed with localized prostate cancer for ∼31 days prior to prostatectomy [16]. Men in the flaxseed arms had significantly lower tumor proliferation rates in prostatectomy tissue. The previous study provided the unique opportunity to explore associations between prostatic ALA, SNPs associated with delta-6 desaturase activity, and biomarkers of aggressive disease e.g., proliferation rate (Ki67) and serum PSA in men consuming a high ALA diet versus regular ALA intake. Our hypotheses were as follows: (1.) We propose that inhibition of ALA metabolism promotes prostate cancer and will lead to positive associations between ALA concentrations in prostatic tissue and serum PSA and tumor proliferation rates; and (2.) We also propose that genetic variation in ALA metabolism modify the relationship between ALA and these biomarkers for aggressive prostate cancer.

## Materials and Methods

### Ethics Statement

All procedures and testing were approved by Duke University Medical Center, the Durham Veteran’s Administration Medical Center and the University of Michigan Community Clinical Oncology Program Institutional Review Boards and written informed consent was obtained prior to all sample and data collection.

### Study Design and Participants

This study utilized data and biological samples from our previous multi-site phase II RCT (NCT00049309) in men with prostate cancer awaiting prostatectomy [16], [^17^]. The RCT included 161 men assigned to control (n = 41), flaxseed (FS) (n = 40), low-fat diet (LF) (n = 40) or FS+LF (n = 40) for ∼31 days prior to surgery. After baseline assessment during which time blood, urine, anthropometric, and medical data were collected, participants were randomized to the study arms based on race (black vs. non-black) and biopsy Gleason sum (<7 vs. ≥7). Regular use of statins and non-steroid anti-inflammatory drugs (NSAIDs) was collected at baseline. As described previously, men assigned to the FS arms were provided with and instructed to consume pre-portioned amounts of flaxseed (30g/d); men in the LF arms consumed <20% of calories from fat; and the control arm was instructed to maintain their usual diet [16]. Recipes and menu suggestions (e.g., mixing it in yogurt, applesauce, or grits) for incorporating flaxseed into the diet were provided to men assigned to these study arms. Follow-up samples were collected within 48 hours prior to surgery (questionnaire data and blood samples) or at the time of surgery (fresh frozen and paraffin-embedded prostatic tissue).

### Dietary Assessment

The NCI Diet History Questionnaire was administered at baseline and follow-up [18]. Data were reviewed by a registered dietitian for logic and completeness. For men assigned to the FS arms, the average amount of flaxseed consumed daily (g/d) was determined based on daily logs kept by the participants. Similar to pill-count methodology, any unused flaxseed was returned and measured [19], [20]. One gram of flaxseed provided 0.057 g and 0.217 g of LA and ALA, respectively. We calculated the total intake of LA and ALA provided by flaxseed supplementation by multiplying the average g/d of flaxseed consumed by the amounts of LA and ALA provided per 1 g of flaxseed. The amount of LA and ALA from flaxseed supplementation was added to values of LA and ALA determined from the DHQ.

### Biological Samples and Assays

Prostatic tissue used for fatty acid analysis was taken from the peripheral zone of the prostate following prostatectomy and flash frozen in liquid nitrogen and stored at −70°C until analyzed. Prostatic tissue fatty acids were analyzed via capillary gas chromatography following tissue homogenization and extraction in chloroform: methanol (2∶1) [21], [22]. The values for prostatic PUFAs are reported as the percentage of total fatty acids identified in prostatic tissue. Using procedures previously described [23], immunohistochemistry was used to determine proliferation index (Ki67 (Biocare, Walnut Creek, CA)) from tissue sections cut from formalin-fixed paraffin-embedded prostatic tumor blocks. Slides were reviewed and scored by two independent pathologists who were blinded to study arm assignment [16].

### *FADS2* Variants and Genotyping

The selection of SNPs was based on the findings of previous investigations in cardiovascular disease that have shown genetic variation to be significantly associated with delta-6 deaturase activity, ALA metabolism and tissue levels of ALA [10], [11], [12], [13], [14]. Based on the current literature, we selected SNPs that correlate with ALA levels in erythrocytes, plasma or serum (rs99780, rs174537, rs174545, rs174572, rs498793, rs3834458, and rs968567) in order to explore the associations between these SNPs and prostatic ALA and prostate cancer biomarkers. These SNPS are located on chromosome 11 within or near the *FADS* gene cluster.

Genomic DNA from whole blood was isolated and purified with the Gentra Puregene Blood Kit (Qiagen, Valencia, Ca). SNPs were genotyped using the pyrosequencing method. Briefly, 20 ng of genomic DNA was amplified with primers specific for each SNP. Primer selection was done using the PSQ Assay design software from Qiagen. A standard PCR reaction was done with 5 PRIME Taq polymerase (Fisher Scientific) consisting of 500 mM KCl, 100 mM Tris-HCl pH 8.3, 15 mM Mg(OAc)_2_, 1% Triton X 100, 0.1 mM each PCR primer and 0.2 mM dNTPs. PCR primers were performed using a touchdown PCR strategy using differing annealing temperatures. All PCR products were checked on a 1.5% agarose gel to ensure amplification and specificity prior to running the pyrosequencing reactions. The pyrosequencing reactions were performed as described by the manufacturer (Qiagen, Valencia, Ca ). Briefly, the resulting biotinylated PCR product was diluted in binding buffer (10 mM Tris-HCL, 2 M NaCl, 1 mM EDTA, 0.1% Tween 20) and bound to sepharose-streptavidin (SA) beads (GE Healthcare, Piscataway, NJ). The dsDNA-SA-beads complex was washed in 70% ethanol, denatured in 0.2 N NaOH and washed in 10 mM Tris-Acetate pH 7.6. The beads were then placed in annealing buffer (20 mM Tris-Acetate, 2 mM MgAc_2_) containing the appropriate sequencing primer (0.3 µM final), heated to 80°C for 2 min and allowed to cool to 25°C. Pyrosequencing was done in the PyroMark HS-96 pyrosequencing machine (Qiagen, Valencia, Ca) as per the manufacturer’s instructions.

### Statistical Analyses

This trial and its statistical analyses were grounded by its 2×2 design; thus flaxseed arms were combined (FS and LF+FS) and compared with the non-flaxseed supplemented arms (control + LF). This allowed a comparison of men consuming a high ALA diet (FS and LF+FS) versus men not consuming a high ALA diet (control + LF). Wilcoxon-signed rank tests and Fisher’s Exact test were used to determine between-group differences in baseline characteristics, dietary and prostatic PUFAs as well as prostate cancer biomarkers. Unadjusted Spearman’s rank correlation coefficients were determined to assess the associations between prostatic PUFAs with PSA and Ki67. To test associations between PSA or log of Ki67 with ALA, linear models were used. In both models, BMI, age, race (black versus non-black), statins or NSAIDS, and flaxseed arm were used as the covariates. The variable ALA was used as a discrete variable and ALA was coded 1 and the absence of ALA was coded as 0. To test the association of PSA and log of Ki67 with the SNPs and their interactions with ALA, the genotype of each SNP and its interaction with ALA were added in the model. Specifically, the dominant model was used due to the small sample size: the most common genotype at each SNP locus was considered as a group (reference group) while the other two genotypes were considered as another group. Therefore, the genotypes of each SNP were coded as 0, 1, and 1 for common homozygote, heterozygote, and rare homozygote genotypes, respectively. The interaction term of SNP genotype and ALA in the regression model was coded as the product of SNP genotype and the ALA. Bonferroni correction was used to adjust for multiple testing. The genotype and allele frequencies of each SNP were estimated and Hardy-Weinberg equilibrium was tested using the chi-square test implemented in the genetics package in R (www.r-project.org/). Using a p-value of 0.001 as suggested by Balding [24], all SNPs were in Hardy-Weinberg equilibrium.

## Results

This investigation included 134 men from the 149 when who completed the original trial. Fresh-frozen tissue was not available following prostatectomy for 15 participants and therefore prostatic levels of PUFAs could not be determined and these participants were excluded from the analysis. Characteristics of the resultant study population are featured in Table 1. We observed no between-group differences in age, racial distribution (black vs. non-black), BMI or Gleason Sum.

**Table 1:** Participant characteristics, dietary and prostatic PUFA levels, and prostate cancer biomarkers (median (range)) among men supplemented with flaxseed for ∼30 days vs. those not supplemented.

	No Flaxseed (n = 67)	Flaxseed (n = 67)	*P*
**Age**	59 (36–71)	60 (44–73)	0.278
**Race, % (n)** **White**	76 (54)	75 (53)	0.846
** Black**	24 (17)	25 (18)	
** BMI**	28.77 (20.95–39.14)	27.28 (20.67–40.72)	0.560
**Statins, % (n)**	28 (20)	24 (17)	0.703
**NSAIDs, % (n)**	39 (28)	38 (27)	1.000
**Gleason Score, % (n)**			
** 5**	3 (2)	6 (4)	0.583
** 6**	43 (29)	39 (26)	
** 7**	46 (31)	51 (34)	
** 8**	6 (4)	1.5 (2)	
** 9**	1 (1)	1.5 (1)	
**Dietary intake of fatty acids g/d** a			
18∶2	11.86 (0.83–55.35)	12.97 (4.47–48.59)	0.813
18∶3	1.23 (0.09–5.65)	7.57 (6.83–11.28)	**<.0001**
20∶4	0.10 (0.01–0.38)	0.09 (0.02–0.67)	0.513
20∶5	0.02 (0–0.25)	0.02 (0–0.25)	0.861
22∶6	0.01 (0–0.08)	0.01 (0–0.13)	0.342
Total omega 3	1.27 (0.09–5.72)	7.66 (6.84–11.35)	**<.0001**
Total omega 6	11.94 (0.84–55.71)	13.05 (4.54–49.26)	0.818
Ratio of 3∶6	0.11(0.05–0.17)	0.60 (0.21–1.55)	**<.0001**
**Prostatic fatty acids**			
18∶2	9.51 (2.76–20.86)	9.35 (0–19.10)	0.141
18∶3	0 (0–0.85)	0 (0–9.41)	0.296
20∶4	0.26 (0.04–1.23)	0.25 (0–1.33)	0.143
20∶5	0.21 (0–1.40)	0.30 (0–1.82)	**0.011**
22∶6	3.58 (0–16.58)	3.92 (0–21.37)	0.460
Total omega 3	3.85 (0.99–17.01)	4.20 (0.44–23.18)	0.227
Total omega 6	9.91 (3.35–20.9)	9.64 (0.75–19.14)	0.231
Ratio of 3∶6	0.40 (0.05–2.19)	0.42 (0.04–14.61)	0.228
**Prostate cancer biomarkers**			
PSA^b^(ng/ml)	5.60 (0.60–14.20)	6.30 (0.5–44.4)	0.056
Ki67	2.93 (0.17–11.10)	1.82 (0.1–13.06)	**0.001**

a No Flaxseed n = 70, Fflaxseed n = 69.

b Flaxseed n = 70.

Dietary intake of PUFAs and PUFA levels in prostatic tissue following flaxseed supplementation are shown in Table 1. No between-group differences were observed for dietary intake of LA, AA and total omega-6 as well as EPA and DHA. However, and as expected, men in the flaxseed arm consumed significantly higher ALA and total omega-3 and therefore had significantly higher ratio of dietary 3∶6 compared to men in the No Flaxseed arm (p<.0001 for all). However, despite the significant differences in ALA intake, we did not observe statistically significant differences in prostatic levels of ALA between the two groups. LA, AA, total omega-6 and total omega-3 were also similar. We noted that the flaxseed arm had significantly higher EPA levels indicating that ALA was converted to EPA in the target tissue. Also included in Table 1 are serum PSA and prostate tumor Ki67. We observed that serum PSA tended to be higher in the flaxseed arm, although this did not reach statistical significance. Consistent with our previous findings, Ki67 was significantly lower in the flaxseed arm compared to those not receiving flaxseed (p = 0.001).

We explored correlations between prostatic PUFAs and prostate cancer biomarkers (Table 2). In unadjusted analyses, prostatic ALA was found to be significantly positively correlated with both serum PSA and Ki67 (ρ = 0.191, p = 0.028 and ρ = 0.0186, p = 0.037). Notably, no other PUFAs correlated with PSA and tumor proliferation rates.

**Table 2:** Correlations between prostatic PUFAs and prostate cancer biomarkers.

	ALA (18∶3)	EPA (20∶5)	DHA (22∶6)	Total omega 3	LA (18∶2)	AA (20∶4)	Total omega 6	Omega 3∶6
**PSA**	**+0.191** a	−0.037	−0.078	−0.060	−0.002	−0.042	−0.003	−0.047
**Ki67**	**+0.186** a	+0.033	−0.088	−0.060	+0.119	−0.059	+0.120	−0.094

a p<0.05.

ALA = Alpha linolenic acid; EPA = eicosapentaenoic acid; DHA = docosahexaenoic acid; Total omega 3 = ALA+EPA+DHA; LA = linoleic acid; AA = arachidonic acid; Total omega 6 = LA+AA; Omega 3∶6 = Total omega 3:Total omega 6.

Based on these analyses, we explored further whether prostatic ALA was independently associated with PSA and tumor Ki67 after adjusting for potential confounders including BMI, age, race, statin drug use, and flaxseed arm (Table 3). In the linear model for PSA, we observed that ALA was positively associated with PSA (p = 0.004) as was flaxseed arm (p = 0.023) and BMI (p<.0001). In the linear model for tumor Ki67, the association with ALA was attenuated but still appeared to be positively associated with higher ki67 (p = 0.051) and the flaxseed arm remained inversely associated with proliferation (p = 0.006). In the same models we explored whether NSAIDs altered the positive association between prostatic ALA and serum PSA and Ki67 (Table S1). In these models, prostatic ALA remained significantly associated with serum PSA (p = 0.004) and remained positively associated with Ki67 although bordering on statistical significance (p = 0.058). NSAIDs were significantly inversely associated with Ki67 (p = 0.017). We also explored dietary intake of LA, prostatic tissue levels of LA, or AA as covariates in this model but these variables were not significant and did not alter the associations between prostatic ALA and PSA or Ki67 (data not shown).

**Table 3:** Independent association between ALA and PSA and Log Ki67.

	Serum PSA		Log Ki67	
Variables	Coefficient	*P*	Coefficient	*P*
**ALA (18∶3)**	**+2.055**	**0.004**	+0.414	0.051
**Flaxseed**	**+1.251**	**0.023**	**−0.452**	**0.006**
**BMI (kg/m^2^)**	**+0.301**	**<.0001**	−0.004	0.859
**Age**	+0.047	0.204	−0.013	0.252
**Race (Non-black)**	+0.428	0.507	+0.287	0.133
**Statin-use**	−0.392	0.176	+0.035	0.684

Because prostatic ALA was positively associated with prostate cancer biomarkers in these previous analyses, we investigated whether SNPs recently shown to influence ALA metabolism were independently associated with prostatic ALA. The distribution of the seven SNPs is shown in Table S2. In these analyses, we did not observe an association between any of the SNPs tested or other covariates with prostatic ALA.

In additional analyses, we explored whether SNPs, prostatic ALA or their interactions were independently associated with PSA or Ki67 (Table 4). In models which included rs174572, rs498793, rs3834458 and rs968567, prostatic ALA was positively associated with PSA (p = 0.002, p<0.001, p = 0.011 and p = 0.033, respectively) and the interaction between rs498793 and prostatic ALA was inversely associated with PSA (p = 0.017). After adjusting for multiple comparisons the significant association between ALA and PSA remained in the models that included rs174572 and rs498793. The SNP, rs174572, was significantly and inversely associated with tumor proliferation rate (p = 0.007). In additional models which included rs99780 and rs174545 significant interactions were found between the SNP and ALA (p = 0.033 and p = 0.047, respectively). After adjusting for multiple comparisons only the model with SNP rs174572 remained statistically significant.

**Table 4:** Associations between ALA, SNPs related to ALA metabolism, and their interaction with PSA and Log Ki67.

		Serum PSA		Log Ki67	
SNP	Variables	Coefficient	*P*	Coefficient	*P*
**rs99780**	**ALA**	+1.205	0.257	−0.078	0.801
	**SNP**	+0.450	0.460	+0.111	0.533
	**Interaction**	+1.572	0.285	**+0.941**	**0.033**
**rs174537**	**ALA**	+1.291	0.166	+0.095	0.728
	**SNP**	+0.310	0.622	+0.076	0.683
	**Interaction**	+1.843	0.216	+0.824	0.071
**rs174545**	**ALA**	+1.281	0.172	+0.059	0.832
	**SNP**	+0.270	0.676	−0.020	0.916
	**Interaction**	+1.881	0.208	**+0.910**	**0.047**
**rs174572**	**ALA**	**+2.242**	**0.002** a	+0.406	0.060
	**SNP**	−0.169	0.891	−**0.453**	**0.007** a
	**Interaction**	−5.010	0.146	N/A^b^	N/A^b^
**rs498793**	**ALA**	**+3.756**	**<0.001** a	+0.332	0.279
	**SNP**	+0.476	0.451	−0.151	0.436
	**Interaction**	−**3.429**	**0.017**	+0.133	0.761
**rs3834458**	**ALA**	**+2.178**	**0.011**	+0.211	0.423
	**SNP**	+0.197	0.759	−0.040	0.842
	**Interaction**	+0.010	0.995	+0.659	0.164
**rs968567**	**ALA**	**+1.658**	**0.033** a	+0.306	0.196
	**SNP**	+0.344	0.635	−0.104	0.636
	**Interaction**	+2.700	0.158	+0.585	0.308

a p<0.05 after Bonferroni correction for multiple testing.

b Due to the low allele frequency of SNP rs174572, there was only sample with ALA and rare homozygote or heterozygote genotype. So the interaction of ALA and SNP rs174572 cannot be added to the model.

## Discussion

This is the first study to explore the impact of feeding an ALA-rich food on levels of fatty acids in the target tissue, and their ultimate association with markers of prostate cancer progression. We found that while flaxseed supplementation resulted in significantly higher levels of ALA in the diet, this consumption did not translate into higher prostatic levels of ALA. This was an important finding because in additional analyses, prostatic ALA was associated with significantly higher PSA and tumor proliferation rates. While the presence of ALA in the prostate appeared to be independent of the SNPs that we investigated, as well as other covariates including dietary LA, we did observe associations between SNPS related to ALA metabolism and interactions between these SNPS and ALA with both PSA and tumor proliferation rates. Thus, findings from this exploratory study provide new evidence to suggest that prostatic ALA metabolism may be associated with aggressive prostate cancer.

PUFA metabolism is complex and the fate of dietary ALA potentially includes β-oxidation, storage in adipose tissues, incorporation into cell membranes and/or desaturation and elongation into longer chain omega-3 PUFAs [25]. In the present study we did not include measures of cellular bioenergetics reflective of the utilization of ALA as a source of energy or storage; however, our data clearly show that higher dietary intake of ALA in the flaxseed arm did not result in higher physiologic levels of ALA in prostatic tissue, but instead resulted in higher prostatic levels of EPA. Given that both study arms had similar dietary intakes of EPA, our data appear to suggest that flaxseed derived ALA was converted to EPA. Interestingly, the higher levels of EPA were not associated with reduced PSA or tumor proliferations rates. These data are in contrast to a recent study which used a similar pre-surgical study design and showed that a low-fat diet supplemented with 5 g/d of EPA resulted in higher prostatic EPA levels and reduced tumor proliferation rates in a subset of men in the RCT [4]. While our study was not able to confirm the anti-proliferative effects of prostatic EPA, we are able to confirm that a high ALA diet results in higher EPA in the target tissue and does not result in accumulation of prostatic ALA.

Several studies have investigated PUFA metabolism in plasma, sera and erythrocytes and have determined that SNPs associated with ALA metabolism play major roles in determining tissue levels of ALA. These SNPs localize near the *FADS* gene cluster at chromosome 11q12–11q13, directly adjacent to a genetic region highly associated with risk for several cancers including prostate cancer [26], [27], [28], [29]. The proximity of these SNPs to the genetic hotspot; the strong association between these SNPS and physiologic levels of ALA; and the observation that physiologic levels of ALA are associated with prostate cancer prompted us to explore whether genetic variation was associated with prostatic ALA and biomarkers indicative of aggressive disease.

In the present study neither dietary intake of PUFAs nor SNPs related to PUFA metabolism explained the presence of prostatic ALA. The discrepancy in findings may be due to the fact that we assayed ALA in the prostates of men with confirmed disease whereas the other studies assayed blood levels and were in healthy populations. This is an important distinction because previous studies have reported that some, but not all, prostate cancer cell lines over-express the LDL receptor compared to normal prostate cells, a characteristic that was also found in a small set of prostate tumors with six out of 12 tumors over-expressing the LDL receptor [30]. Similar findings have been reported in colon cancer [31]. Because LDL is the primary vehicle for delivery of ALA to peripheral tissues, the over-expression of the LDL receptor in some prostatic tumors may increase the transfer of ALA to the tissue.

We also observed that four out of the seven SNPs tested, were significantly related to either PSA or tumor proliferation rates either independently or through an interaction with prostatic ALA. However, not all of these associations remained after adjusting for multiple comparisons likely due to our limited sample size. For example, we observed positive associations with tumor proliferation for the interactions between prostatic ALA and the presence of the minor allele in the SNPs rs99780 and rs174545, as well as an inverse association with PSA for the interaction between prostatic ALA and the presence of the minor allele in the SNP rs498793. Previous studies have established that the presence of the minor allele in all of the SNPs tested was associated with higher blood levels of ALA [7]–[11]. In the present study, while we did not observe that these SNPs were independently associated with prostatic ALA, we did observe that some of them modified the association between prostatic ALA and PSA or tumor proliferation. A potential explanation for this finding relates to tissue-specific differences in gene expression of desaturase enzymes [8], [^32^]. Consistent with this hypothesis, it has been reported that SNPs have differential effects on gene expression within different tissue sources suggesting that tissue-specific splice variants play an important role in determining gene expression [33].

Our study confirms the observations of Christensen et al. who reported that men with prostate cancer had higher ALA levels compared to men with benign prostate hyperplasia [34]. Similar to our findings, Christensen and colleagues also observed a significant correlation between prostatic ALA and PSA; however, they did not measure tumor proliferations rates. Some potential mechanisms linking higher prostatic ALA with more aggressive prostate cancer may be through increased activation of cell signaling pathways. Previous studies have reported that ALA induces gene expression of MEK1 and MEKK1 which can stimulate transcriptional activity of the androgen receptor [35], [36]. Further, to our knowledge, no study has determined the associations between SNPs related to ALA metabolism and delta-6 desaturase gene expression in prostatic tissue. Based on our findings, additional studies are warranted and may elucidate the associations that we observed between SNPS and prostate cancer biomarkers.

As with all studies, this study has strengths and limitations. One imitation is the relative short time frame that subjects consumed a high ALA diet and the absence of baseline fresh-frozen prostatic tissue to assess PUFA changes in the target tissue. We acknowledge that this study is a secondary analysis and that the original study was not designed to test the hypothesis explored in the current investigation. Moreover, we did not replicate our exploratory findings in an independent population. Therefore, future studies with larger sample sizes are needed to confirm these findings. However a key strength of this study is that data and biological samples emanated from one of the largest presurgical trials in prostate cancer patients to date. Moreover, attrition was minimal and adherence to the protocol was excellent [16]. This study also is one of the few that has provided dietary intake, as well as direct physiologic measurements of PUFAs in the target tissue and explored their associations with genetic factors and biomarkers indicative of disease course.

In summary, this study showed that among prostate cancer patients, prostatic ALA, independent of diet, was significantly and positively associated with biomarkers of aggressive disease, i.e., both higher PSA and tumor proliferation rates. We also found novel evidence that genetic variation related to ALA metabolism influences the association between ALA and prostate cancer biomarkers. Thus, the results of this exploratory study suggest a gene-nutrient interaction that may be an important mediator of aggressive prostate cancer, and one that could be exploited to discriminate indolent vs. virulent disease. Future studies are needed to confirm these findings.

## Supporting Information

Table S1 **Independent association between ALA and PSA and Log Ki67.**
(DOCX)

Table S2 **Distribution of SNPs among study participants.**
(DOCX)
