# Supplementary material for: Prostatic Alpha-Linolenic Acid (ALA) Is Positively Associated with Aggressive Prostate Cancer: A Relationship Which May Depend on Genetic Variation in ALA Metabolism
Source: PLoS One. 2012 Dec 28;7(12):e53104. doi: 10.1371/journal.pone.0053104 (PMC3532426; doi:10.1371/journal.pone.0053104)
Supplement: Table S1 — Independent association between ALA and PSA and Log Ki67. (DOCX) [file pone.0053104.s001.docx]

| **Table S1 Independent association between ALA and PSA and Log Ki67** | | | | |
| --- | --- | --- | --- | --- |
|  | **Serum PSA** | | **Log Ki67** | |
| **Variable** | **Coefficient** | ***P*** | **Coefficient** | ***P*** |
| **ALA (18:3)** | **+1.008** | **0.004** | +0.196 | 0.058 |
| **Flaxseed** | **+0.616** | **0.025** | **-0.221** | **0.006*** |
| **BMI (kg/m^2^)** | **+0.290** | **<.0001*** | -0.002 | 0.935 |
| **Age** | +0.041 | 0.268 | -0.012 | 0.269 |
| **Race (Non-black)** | -0.145 | 0.652 | -0.158 | 0.095 |
| **NSAIDS** | -0.334 | 0.232 | **-0.194** | **0.017*** |
