# Supplementary material for: Prostatic Alpha-Linolenic Acid (ALA) Is Positively Associated with Aggressive Prostate Cancer: A Relationship Which May Depend on Genetic Variation in ALA Metabolism
Source: PLoS One. 2012 Dec 28;7(12):e53104. doi: 10.1371/journal.pone.0053104 (PMC3532426; doi:10.1371/journal.pone.0053104)
Supplement: Table S2 — Distribution of SNPs among study participants. (DOCX) [file pone.0053104.s002.docx]

| **Table S2. Distribution of SNPs among study participants** | | | |
| --- | --- | --- | --- |
| **SNP** | **Allele** | **Genotype** | **HWE**  **p-value** |
| **rs99780** | G 196 67%  A 98 33% | G/G 70 48%  G/A 56 38%  A/A 21 14% | 0.094 |
| **rs174537** | G 225 77%  T 69 23% | G/G 92 63%  G/T 41 28%  T/T 14 10% | 0.010 |
| **rs174545** | G 222 76%  C 72 24% | G/G 90 61%  G/C 42 29%  C/C 15 10% | 0.007 |
| **rs174572** | C 282 97%  T 10 3% | C/C 138 95%  C/T 6 4%  T/T 2 1% | 0.007 |
| **rs498793** | G 175 60%  A 117 40% | G/G 57 39%  G/A 61 42%  A/A 28 19% | 0.122 |
| **rs3834458** | T 225 78%  - 65 22% | T/T 94 65%  T/- 37 26%  -/- 14 10% | 0.003 |
| **rs968567** | C 261 89%  T 33 11% | C/C 116 79%  C/T 29 20%  T/T 2 1% | 1.0 |
